# Implementation of Novel Design Features for qPCR-Based eDNA Assessment

**DOI:** 10.1371/journal.pone.0164907

**Published:** 2016-11-01

**Authors:** Nik Veldhoen, Jared Hobbs, Georgios Ikonomou, Michael Hii, Mary Lesperance, Caren C. Helbing

**Affiliations:** 1 Department of Biochemistry and Microbiology, University of Victoria, P.O. Box 3055, STN CSC, Victoria, British Columbia, V8W 2Y2, Canada; 2 Hemmera Envirochem Inc., 303–1221 Broad Street, Victoria, British Columbia, V8W 2A4, Canada; 3 Department of Mathematics and Statistics, 3800 Finnerty Road, University of Victoria, Victoria, British Columbia, V8P 5C2, Canada; Oklahoma State University, UNITED STATES

## Abstract

Environmental stewardship requires timely, accurate information related to the status of a given ecosystem and the species that occupy it. Recent advances in the application of the highly sensitive real-time quantitative polymerase chain reaction (qPCR) towards identification of constituents within environmental DNA (eDNA) now allow targeted detection of the presence of species-specific biological material within a localized geographic region. However, as with all molecular techniques predicated on the specificity and sensitivity of the PCR assay, careful validation of each eDNA qPCR assay in development must be performed both under controlled laboratory conditions and when challenged with field-derived eDNA samples. Such a step-wise approach forms the basis for incorporation of innovative qPCR design features that strengthen the implementation and interpretation of the eDNA assay. This includes empirical determination that the qPCR assay is refractory to the presence of human DNA and the use of a tripartite assay approach comprised of 1) a primer set targeting plant chloroplast that evaluates the presence of amplifiable DNA from field samples to increase confidence in a negative result, 2) an animal group primer set to increase confidence in the assay result, and 3) a species-specific primer set to assess presence of DNA from the target species. To demonstrate this methodology, we generated eDNA assays specific for the North American bullfrog (*Lithobates (Rana) catesbeiana*) and the Rocky Mountain tailed frog (*Ascaphus montanus*) and characterized each with respect to detection sensitivity and specificity with demonstrated performance in a field survey scenario. The qPCR design features presented herein address specific challenges of eDNA assays thereby increasing their interpretative power.

## Introduction

In the realm of environmental science, an understanding that genetic material can be harvested from ecosystem biota has been known for decades [1]. Recent technical advances in polymerase chain reaction (PCR) or its more recent variation, quantitative real-time PCR (qPCR), now allow for the successful detection of minute amounts of species-specific nuclear or mitochondrial DNA material in collected aquatic or terrestrial/sediment field samples with the application of qPCR methodology [2–6]. Such analysis of ‘environmental DNA’ or eDNA is being used to augment traditional field survey techniques of macrobiota and often allows for increased accuracy in our understanding of native or introduced species. These methods can be used to efficiently refine existing information pertinent to conservation biology, dispersal of invasive species, and management of industrial impacts on the environment [7–11]. This method is cost-effective, non-invasive to the organism and habitat, and can be highly sensitive in a wide range of habitats. This is particularly invaluable relative to traditional survey techniques for rare or elusive species which are often labour-intensive and inefficient, and, as mentioned above, destructive to organismal habitats. In contrast, eDNA can be measured from a simple water sample without disturbing the species of interest which is then filtered. DNA is then isolated from the filter and analyzed using qPCR methods. Because of its sensitivity, eDNA methods are best used for target species that are present at low density exactly under those conditions where traditional methods are least effective. Another advantage of eDNA methods is in the ability to archive samples indefinitely and query them multiple times for species of interest as need arises.

Due to its powerful sensitivity, establishment of appropriate rigour in the design and performance of the eDNA assay must be maintained to reduce the likelihood of false positive and negative results (i.e., Type 1 and Type 2 errors, respectively) that could impact the quality of management decisions based upon, for example, the successful eradication of invasive species or determination of presence or absence of endangered species. Considerable effort has been applied to critical aspects in the design and execution of eDNA methods and an extensive overview of the current state-of-the-art along with guidelines and considerations for implementing eDNA methods has recently been published [12]. The greatest concentrated effort has been focused upon methods pertaining to sample collection in the field, DNA extraction, and issues regarding data interpretation pertaining to eDNA distribution and dynamics in the environment. However, comparatively little detailed attention and clarity has been given to the actual qPCR assay design.

Detection of eDNA relies completely upon the qPCR assay which is often pushed to the limit in specificity and sensitivity requirements. The approach that has been taken to date [12] has focused upon development of species-specific primer sets through three design steps: 1) *in silico*, 2) *in vitro*, and 3) *in situ*. *In silico* design involves computer-based alignment of known DNA sequences from target and non-target species (when known) to identify regions that may represent discriminatory priming locations for qPCR-based detection of the target species. Selected primer set candidates are then tested on DNA from target and non-target species *in vitro* to evaluate assay specificity. Finally, *in situ* verification involves applying the primer set candidates that have passed the *in vitro* design step to eDNA samples obtained from environments with confirmed presence and absence of the target species. This latter step should be incorporated in every eDNA study as important assay controls.

Despite acknowledgment of these important qPCR assay design aspects, there remains limited clarity on other important design, validation, and execution criteria that must be considered as they greatly influence the interpretive power of the qPCR portion of the eDNA assay. These include: explicitly designing and testing qPCR assays against detection of human DNA that may be introduced at the sampling and/or analysis stages; additional aspects of primer and probe design considerations; qPCR run conditions to enhance specificity based upon biochemical principles; the ability to distinguish between true and false negatives through evaluation of endogenous DNA found in all field-collected eDNA samples rather than through the laboratory-based practice of spiking samples with an external DNA template; and, the ability to enhance confidence in the assay result through the use of an animal group-based qPCR assay.

With escalation in the application of eDNA-associated detection assays the present initiative addresses the need for enhancement in the considerations required for design, validation, and execution of a qPCR-based eDNA method. To this end we provide specific examples from the North American bullfrog (*Lithobates* (*Rana) catesbeiana*; hereafter referred to as bullfrog), and the Rocky Mountain tailed frog (*Ascaphus montanus*; hereafter referred to as tailed frog). Both species are listed as least concern by the International Union for Conservation of Nature (IUCN; https://www.iucn.org/resources/conservation-tools/iucn-red-list-threatened-species), although there are some populations that are known to be at risk. The bullfrog is a species that is native to eastern North America and has been introduced to the west and all continents except Antarctica worldwide. Some native bullfrog populations (such as in eastern Ontario and Québec) are threatened whereas introduced populations, such as those in British Columbia, may contribute to declines of other native frog species not adapted to their presence. Despite its high abundance in the Northwestern United States, the tailed frog is federally designated as threatened by the Committee on the Status of Endangered Wildlife In Canada (COSEWIC; http://www.cosewic.gc.ca/eng/sct5/index_e.cfm) and a red-listed species in British Columbia due to a restricted range, low number of known occurrence records, low population size in geographic locations, and stream sedimentation due to roads, logging and fire.

Using these two species as examples, we present a novel hierarchical approach for qPCR assay design specifically for eDNA applications using water samples and filtration techniques. The development and evaluation of such a hierarchical validation process will help mitigate potential for false positive and false negative results to provide greater confidence in eDNA-based methods and improved interpretive power in the associated results.

## Materials & Methods

### Collection and Filtration of Environmental Water Samples

Duplicate environmental water samples were collected from the water’s edge at six locations on either Vancouver Island for the assessment of bullfrog presence or the thalweg of lotic systems in Southeastern British Columbia, Canada for the assessment of tailed frog presence. All locations were on public land with unrestricted access and the collection of water samples did not require specific permissions. Water samples (250 mL for bullfrog testing or 1000 mL for tailed frog testing) were collected at each field location in polypropylene bottles and stored in a cooler with ice packs during transport. All water samples underwent filtration within 24 hours of collection.

For the bullfrog test locations, two reusable Swinnex filter holders containing O-rings (Millipore Ltd., Etobicoke, ON, Canada; Cat# SX0002500) were prepared with one containing a Whatman 24 mm diameter glass microfibre filter (GE Healthcare Life Sciences, Mississauga, ON, Canada; Cat# 1827–024) and the other containing a MF-Millipore 25 mm mixed cellulose membrane filter (0.45 μM pore size; Millipore; Cat# HAWP02500). Each water sample was loaded in multiple aliquots into a 60 mL BD Luer-Lok tip syringe (VWR International, Mississauga, ON, Canada) which was then attached in serial to first the glass filter and then the cellulose membrane filter. The entire water sample was filtered through the assemblage with repeated reloading of the syringe and a replacement of the Swinnex containing the glass filter, if required, to maintain an adequate flow rate. The Swinnex filter holders were reused for additional sample filtration once treated with bleach (5% w/v sodium hypochlorite) for 15 minutes followed by a 10 minute rinse with distilled water and air drying. For the tailed frog test locations, 1000 mL of water was collected and filtered under vacuum through a 47 mm cellulose nitrate filter (0.45 μM pore size; Thermo Fisher Scientific Inc., Ottawa, ON, Canada; Cat# N1452045). For all survey sites sampled, following filtration, cellulose filters were removed and placed individually in 2 mL screw-cap tubes containing 95–100% molecular grade ethanol.

### Isolation of Total DNA from Filtered Water Samples

Prior to isolation of total DNA, all sample filters were randomized and processed blind to eliminate bias. All further work with filter samples was performed in a biological safety cabinet (NuAire, Plymouth, MN, USA) including total DNA isolation from filters and assembly of the eDNA qPCR assay. In all instances of handling individual filter samples, non-serrated stainless steel forceps were used that had been treated sequentially with a bleach (5% w/v sodium hypochlorite) wash, a distilled water rinse, and then wiped dry. After transport to the lab, filters were removed from ethanol, air dried on Whatman 55 mm diameter filter paper, and halved using the forceps. One half filter was placed in a new 1.5 mL tube for DNA isolation while the remaining unprocessed half filter was returned to 95–100% ethanol. Total DNA was recovered from each filter sample using the DNeasy Blood and Tissue Kit (QIAGEN Inc., Mississauga, ON, Canada; Cat# 69506) as per the manufacturer’s protocol with the following modifications; samples to be tested for the presence of bullfrogs used 180 μL Buffer ATL while samples to be tested for tailed frogs required 280 μL Buffer ATL and the remaining reagents were adjusted proportionally. For each sample, following overnight proteinase K digestion, both filter and liquid were transferred to a DNeasy column using bleach-treated and distilled water-rinsed forceps. DNA was eluted from each column with 100 μL Buffer AE. DNA was eluted from the spin column with 100 μL of Buffer AE and the nucleic acid concentration determined by A_260_ spectrophotometry. DNA samples were stored at -20°C prior to use in the eDNA qPCR assay.

### DNA Primer Design

TaqMan-based primer sets directed towards frogs were obtained from previously published information (eLICA1 [13] and eASMO [14]) or designed using organelle gene-specific sequence data from multiple species (NCBI Genome, http://www.ncbi.nlm.nih.gov/genome/organelle/). An analogous generation of primer sets was carried out on chloroplast gene sequences obtained from freshwater plant species. Table 1 identifies the mitochondrial and chloroplast gene sequences and their associated or confounding species used in primer development. For each gene target, species-specific sequences were aligned by ClustalW (http://www.genome.jp/tools/clustalw/) and the output aln file assessed using BioEdit (Ibis Biosciences, Carlsbad, CA, USA) and Primer Premier version 5 (Premier Biosoft, Palo Alto, CA, USA) for generation of either cross-species (ePlant5 and eFrog2, 3, and 5) or species-restricted (eLICA2 and eASMO9) primer sets. Note that the eLICA1 and eASMO hydrolysis probes were extended by four bases in the 3’ direction from that previously published [13,14] in order to allow for use of an increased annealing temperature to enhance stringency in the present eDNA assay (Table 2). DNA amplification primers and associated TaqMan hydrolysis probes were obtained from Integrated DNA Technologies (Coralville, IA, USA) and their characteristics are shown in Table 2.

**Table 1:** Cross-species gene sequence information incorporated into eDNA primer set design^a^.

Primer Set	ePlant5	eFrog2, eFrog3, eFrog5
Gene Target	chloroplast 23S rRNA (*rrn23*)	mitochondria 16S rRNA (*mtrnr2*)
Comparator	*Paradoxia multiseta*; KM462879	*Lithobates catesbeiana*: M57527
Species^b^	*Chlorella vulgaris*: AB001684	*Lithobate sylvatica*: AB639591
	*Chlamydomonas reinhardtii*: FJ423446	*Lithobates clamitans*: AY779204
	*Scenedesmus obliquus*: DQ396875	*Rana luteiventris* AY779194
	*Staurastrum punctulatum*: AY958085	*Ascaphus truei*: AJ871087
	*Closterium baillyanum*: KF060940	*Pseudacris regilla*: AY291112
		Pseudacris maculate; AY291089
		Pseudacris illinoensis: AY291110
		*Homo sapiens*: AP008824
Primer Set	eLICA2	eASMO9
Gene Target	mitochondria 12S rRNA (*mtrnr1*)	mitochondria cytochrome B (*cytb*)
Comparator	*Lithobates catesbeiana*: M57527	*Ascaphus montanus*: DQ087517
Species^b^	*Lithobates sylvatica*: KP222281	*Ascaphus truei*: AF277330
	*Rana maculata*: DQ283303	*Lithobates clamitans*: AY083277
	*Rana temporaria*: AB685766	*Lithobates catesbeiana*: NC_022696
	*Lithobates pipiens*: DQ283123	*Lithobates sylvatica*: NC_027236
	*Pseudacris regilla*: AY819376	*Rana aurora*: EU552219
	*Pseudacris crucifer*: AY819385	*Pseudacris maculata*: KJ536217
	*Bufo americanus*: AY680211	*Pseudacris crucifer*: KJ536191
	*Spea bombifrons*: AY819327	*Pseudacris regilla*: KJ536196
	*Ascaphus truei*: AJ871087	*Bufo americanus*: AB159264
	*Homo sapiens*: AP008824	*Bufo boreas*: HM563929
		*Homo sapiens*: AP008824

^a^Selection of pertinent species for use in eDNA primer design was performed using information on anurans from the Canadian Biodiversity (http://canadianbiodiversity.mcgill.ca/english/species/herps/anura.htm) and the BC Frogwatch Program (http://www.env.gov.bc.ca/wld/frogwatch/) websites and for freshwater plants using the Canadian Center for the Culture of Microorganisms (http://www3.botany.ubc.ca/cccm/FWAC/fwaccatalog.html) and AlgaeBase (http://www.algaebase.org/search/distribution/) websites.

^b^Each selected comparator species is followed by the NCBI GenBank accession number of the relevant sequence used in primer set design.

**Table 2:** Characteristics of PCR primers evaluated in the development of eDNA assays for bullfrog and tailed frog, cross-species plant probe, and a cross-species frog probe.

Species Target	Gene Target	Primer Set	Primer Name	Primer Sequence	Amplicon Size	Reference
xPlant	chloroplast 23S rRNA	ePlant5	150134F	TCTAGGGATAACAGGCTGAT	147	Present work
			150135R	TGAACCCAGCTCACGTAC		
			150139 Probe	TTTGGCACCTCGATGTCGG		
xFrog	mitochondrial 12S rRNA	eFrog2	150013F	AGGYGGATTTAGYAGTAAAAAG	155	Present work
			150014R	TAYACTTACCATGTTACGACTT		
			150050 Probe	ACACACCGCCCGTCACCCTC		
xFrog	mitochondria 16S rRNA	eFrog3	150015F	GGAAAGRTGAAATAGAAATGAAA	142	Present work
			150016R	GTAGCTCRCTTAGTTTCGGG		
			150051 Probe	TCGTACCTTTTGCATCATGGT		
xFrog	mitochondrial 16S rRNA	eFrog5	150019F	AGTTACCCTRGGGATAACAG	121	Present work
			150020R	AACAAACGAACCWTTAGTAGC		
			150053 Probe	TTTACGACCTCGATGTTGGATCAG		
LICA	mitochondria cytochrome B	eLICA1	BullfrogF	TTTTCACTTCATCCTCCCGTTT	84	Strickler et al., 2015
			BullfrogR	GGGTTGGATGAGCCAGTTTG		
			Bullfrog Probe^a^	TTATCGCAGCAGCAAGTATGA		
LICA	mitochondria 12S rRNA	eLICA2	150003F	GAGAACGCCCTTTAAATCTT	135	Present work
			150004R	GTCAAGCTGACGCTCATACG		
			150046 Probe	ACAAACCCTCCGCCCACAAC		
ASMO	mitochondria cytochrome B	eASMO	qASMOF	ACGTCAACTATGGCTGGCTAATC	90	Pilliod et al., 2013
			qASMOR	GTCCTCGGCCAATGTGAAGA		
			ASMOProbe^a^	CATGCAAATGGAGCATCATTC		
ASMO	mitochondria cytochrome B	eASMO9	150151F	ACTTTATTACGGCTCTTACTTG	176	Present work
			150153R	GTACGTTTCCGATGTAAGGGA		
			150169 Probe	ATACGTATTACCATGAGGACAAATATC		

^a^Primer sequences used in this work are modified from that previously published [13,14] by addition of 4 additional bases at the 3’ end to satisfy more stringent annealing conditions.

### Isolation of Total DNA from Animal Tissues for qPCR Primer Validation

Total DNA for qPCR primer specificity and sensitivity validation was isolated from confirmed species sources: bullfrog tadpole tail muscle, *Lithobates (Rana) pipiens* (Northern leopard frog) adult liver, *Pseudacris* regilla (Pacific tree frog) tadpole whole body, tailed frog tadpole tail muscle, and *Xenopus laevis* (South African clawed frog) tadpole heart as well as from *Homo sapiens* (human) HEK293 cells using the DNeasy Blood and Tissue Kit (QIAGEN) with inclusion of RNase treatment as described by the manufacturer. Tissues were taken under the appropriate sanctioned protocols and permits approved by the University of Victoria Animal Care Committee (Protocol #2015–028) and British Columbia Ministry of Forests, Lands and Natural Resource Operations (MFLNRO) permit VI11-71459. One bullfrog and Pacific tree frog tadpole each were locally caught, one adult Northern leopard frog was obtained from Carolina Biological Supply Company (Burlington, NC), and the *Xenopus laevis* tadpole was bred at the University of Victoria Aquatics Facility. Tailed frog tadpole tail muscle preserved in ethanol was obtained from a tadpole that died of natural causes under MFLNRO permit MRNA 15–170593.

Live animals were euthanized using 0.1% w/v (tadpoles) or 1% w/v (adults) tricaine methane sulfonate in dechlorinated municipal water containing 25 mM sodium bicarbonate prior to tissue collection. DNA was eluted from the spin column with 150 μL of Buffer AE and the nucleic acid concentration determined by A_260_ spectrophotometry. DNA samples were stored at -20°C prior to use in the eDNA qPCR assay.

### Validation of qPCR Primer Sets for Application towards eDNA Detection

The selectivity and sensitivity of bullfrog (eLICA1 and eLICA2), tailed frog (eASMO and eASMO9) and the cross-species frog (eFrog2, 3, and 5) primer sets were examined along with the efficacy of the plant chloroplast detection primer set (ePlant5) on an Mx3005P qPCR system (Agilent Technologies Inc., Santa Clara, CA, USA). Each 15 μl qPCR amplification reaction consisted of 10 mM Tris-HCl (pH 8.3 at 20°C), 50 mM KCl, 3 mM MgCl_2_, 0.01% Tween 20, 0.8% glycerol, 69.4 nM ROX (Life Technologies, Burlington, ON, Canada), 10.5 pmol of forward and reverse PCR primer, 1.5 pmol of TaqMan hydrolysis probe, 200 μM dNTPs (FroggaBio Inc., North York, ON, Canada), one unit of Immolase DNA polymerase (FroggaBio), and 2 μl of DNA sample. The qPCR assembled to evaluate the ePlant5 primer set included 2 μL of isolated DNA sample from field sites 1, 6, and 7 as well as from tap water. In the selectivity test, the DNA sample included in the qPCR reaction was 2 μL of 5 μg/L isolated total DNA from bullfrog, leopard frog, tree frog, clawed frog, tailed frog, or human.

The sensitivity test included 2 μL addition of a five-fold dilution series (0.008–5 μg/L) of bullfrog (for eLICA1 and eLICA2) or tailed frog (for eASMO and eASMO9) isolated total DNA plus a “no DNA template” negative control. Twenty-three (ASMO) or twenty-five (LICA) technical replicates were run for each filtered water sample to determine the binomial error range at each dilution. Technical replicates involve running a replicate qPCR reaction on the same filtered eDNA sample. Binomial standard error was calculated as the square root of the product of the proportion of positives and negatives divided by the total number of technical replicates (Table 3).

**Table 3:** Effect of technical replicates on binomial standard error of all of the animal qPCR primer sets evaluated on 0.04 μg/L of total DNA isolated from the indicated species.

	Binomial standard error^a^ range	
Number of technical replicates	Bullfrog	Tailed frog
23 or 25^b^	0.07–0.09	0.09–0.10
12	0.11–0.13	0.12–0.14
8	0.13–0.16	0.15–0.18
3	0.21–0.27	0.24–0.29

^a^Binomial standard error = $\sqrt{(proportion\;positive\;x\;proportion\;negative)/n}$

^b^For tailed frog and bullfrog, respectively

DNA amplification reactions were subject to the following thermocycle conditions: an initial activation step of 9 min at 95°C followed by 50 cycles of 15 sec denaturation at 95°C, 30 sec annealing at 64°C, and 30 sec polymerization at 72°C. Sequence specificity in qPCR-mediated DNA amplification for each primer set was confirmed through restriction endonuclease mapping of the DNA product.

### Field Application of qPCR-Based eDNA Assays

Eight TaqMan-based qPCR replicate reactions were performed for each field-derived sample and carried out as described above. Appropriate assay performance was determined for each qPCR plate by inclusion of a negative control lacking addition of DNA template and a positive control that contained 5 μg/L target-specific total DNA. Individual qPCR reactions were scored as positive if DNA amplification occurred within 30 (ePlant5) or 50 cycles (eLICA1, eASMO9, and eFrog3).

## Results and Discussion

Development of a PCR-based assay that serves to detect the presence of a single animal species or a select group of related animals from a complex environmental DNA source is no small feat. In addition to appropriate sample collection methodology, an ‘informed design’ must be performed for the molecular assay whereby known evolutionarily closely related species, members of the same animal family that are situated in the geographic survey region, and similar human sequences to the target gene are all factored into the primer design process in an attempt to maximize eDNA assay specificity towards the target species and/or more broadly towards a given family or genera.

As the target biological material is likely to be present at low abundance in field collected water samples, a number of factors are included in the eDNA assay. First, gene sequences used in assay development are commonly sourced from the mitochondrial genome, as the number of copies of this DNA template disseminated into the surrounding environment from a given species is higher in comparison to the nuclear genome. An additional consideration is the evolutionary plasticity that may be inherent in the nuclear genome across subspecies whereby whole genome duplication, gene fractionation, and pseudogenes lend increased complexity in DNA sequence targeting [15]. While potentially advantageous for inspection of the geographic distribution of a specific subspecies, such variation in the nuclear genome can confound evaluation at higher taxonomic levels (e.g. species, genus, or family). While the present work is centered upon analysis of eukaryotic species, survey of prokaryotic and viral populations within a given ecosystem can also be undertaken bearing in mind that diligent application of experimental controls are required during assay validation to exclude the influence, on eDNA assay development, of contaminated commercial reagents that may be used in nucleic acid isolation and PCR-based amplification [16–18].

### Species Specificity of eDNA Assay Tools

The first phase of eDNA assay development involves *in silico* informatics to inform gene-specific primer design. The present eDNA assay includes two formats for detection of vertebrate species. The first format is designed to detect specific target species. The second involves a cross-species primer set capable of detecting multiple species representing an animal group.

Available information on mitochondrial gene sequences for the anuran target species and related potential confounder species were evaluated through DNA alignments. Candidate primer sets in this instance were either obtained from previously published studies (eLICA1 and eASMO) or generated (eLICA2 and eASMO9) for bullfrog and tailed frog, respectively. Three cross-species primer sets capable of detecting multiple frog species representing the animal group (eFrog2, eFrog3, eFrog5) were also designed *de novo*. Each of these primer sets were designed to eliminate possible cross-reaction with human mitochondrial DNA.

Table 2 demonstrates that selection of a certain mitochondrial gene over other candidates is not particularly advantageous and that all design possibilities to generate the eDNA assay should be empirically evaluated for their efficacy in PCR. Secondly, the PCR thermocycle is extended beyond its usual limits in an eDNA assay and DNA sequence (hence species) specificity is preserved with inclusion of TaqMan-associated detection in a qPCR format. The presence of both the DNA amplification primer pair and a hydrolysis probe in assay development serves to enhance target specificity by allowing for increased placement of cross-species base-pair mismatches that minimize false positive detection of related members of the animal family or genus [19].

**Fig 1:**
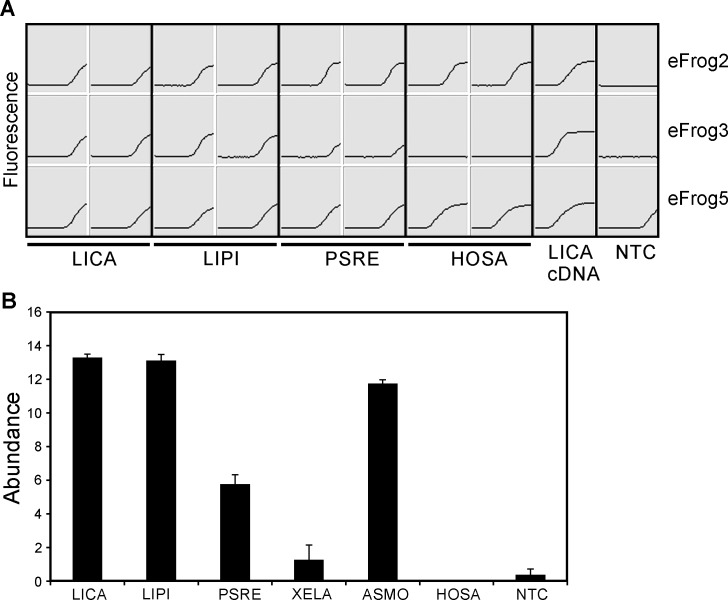
Cross-species analysis of qPCR-based detection of mitochondrial gene sequence with primer sets designed towards anurans. A) Fluorescent-based amplification curves are shown representing assay reactions assembled using TaqMan-associated qPCR primer sets eFrog2, eFrog3, or eFrog5 and total DNA template from bullfrog (LICA), leopard frog (LIPI), tree frog (PSRE), or human (HOSA). Duplicate eDNA qPCR assay reactions are shown. A positive control comprising bullfrog brain cDNA (LICA cDNA) as well as a no template negative control (NTC) were included in the assay. For each reaction, successful amplification of DNA is shown by an increasing fluorescent signal. B) Graphical representation of eFrog3 amplification results of replicate qPCR reactions (n = 23 to 27) performed for against each DNA template with the mean abundance and standard error of the mean shown. Abundance for each qPCR replicate was determined as the assay thermocycle limit (50) minus the cycle threshold (C_t_) value when amplification was detected.

The extended thermocycle program necessary for evaluation of eDNA requires application of PCR-clean practices to minimize sample cross contamination and maintain eDNA assay integrity [20]. eDNA assay design pushes the limits of PCR detection and involves determination of an acceptable balance between sensitive, targeted detection and minimization of false positives for each individual test performed. One constant in all eDNA assessment strategies is involvement of humans in sample collection, processing, and evaluation. Thus, an absolute requirement during methods development is confirmation that the eDNA assay does not detect human biological material including that of the field biologist collecting the sample and the laboratory personnel carrying out the qPCR assay as these represent a potential source of false positive signal. *In silico* analysis is not the final determining factor in ensuring a lack of unintentional cross-species priming. Rather an additional empirical evaluation must be made as demonstrated in our experience with designing frog cross-species primer sets. Although all three eFrog primer pairs amplified frog DNA very well, two of the TaqMan-based primer sets also detected human DNA (Fig 1A). Thus, caution must be used in relying solely on *in silico* design parameters for determination of primer efficacy; undertaking a robust empirical validation program is essential.

### Determination of Specificity of eDNA Assay Tools

Evaluation of eDNA primer set efficacy includes determination of both maximum detection specificity and sensitivity with an absolute requirement for the former parameter prior to investigation of the latter. While not an exact replacement for the complexity inherent in field-derived eDNA samples, use of serially-diluted purified total DNA from the species under consideration to define the extent of signal capture at low DNA template amounts is a reasonable course of action [21].

We support application of a three-step thermocycle in lieu of the more frequently used two-step method [5,14,19,22] and the use of stringent annealing temperatures during empirical assessment of DNA primer sets in an attempt to maximize specificity by exploiting the limited base pair mismatches that may exist within target genes between closely related species. In addition to eDNA tools that identify a single species, an opposing primer design criteria should be undertaken that exploits conserved sections of the target gene sequence to generate cross-species capability.

**Fig 2:**
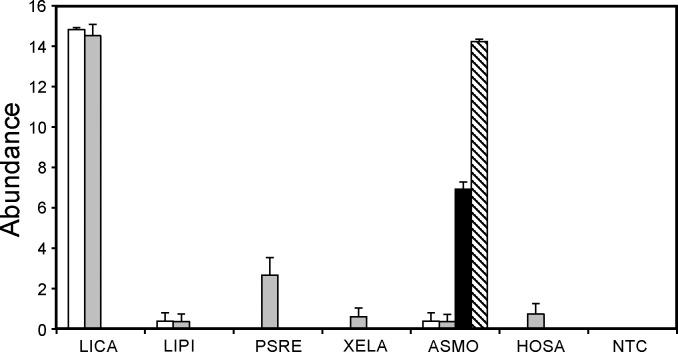
Selectivity in detection following development of species-specific qPCR primer sets. The species-specific primer sets included eLICA1 (white bar), eLICA2 (grey bar), eASMO (black bar), and eASMO9 (hatched bar). DNA template included in species-specific amplification reactions comprised bullfrog (LICA), leopard frog (LIPI), tree frog (PSRE), clawed frog (XELA), tailed frog (ASMO), and human (HOSA). Additionally, all primer sets were evaluated in a qPCR reaction with no DNA template present (NTC). Multiple qPCR reactions (n = 23 to 27) were performed for each primer set and DNA template combination with the mean abundance and standard error of the mean determined and presented as described in Fig 1.

*In silico* designed primer sets were next evaluated through a laboratory validation pipeline. Both eLICA1 and eLICA2 primer sets preferentially detected bullfrog total DNA compared to the other frog species investigated (Fig 2). However, eLICA2 also demonstrated moderate amplification from tree frog and weak detection of clawed frog and human total DNA leading to reduced species specificity. The primer sets associated with tailed frog (eASMO and eASMO9) displayed strong detection of the target species total DNA with no cross-species reactivity (Fig 2).

The frog group primers, eFrog2, 3, and 5 were specifically designed to work for many phylogenetically distinct frog species and to not amplify human-derived DNA. Initial evaluation showed a strong amplification signal for each of these primers. Each primer set showed detection of all five frog species tested (Fig 1A). However, eFrog3 primers were the only ones that did not amplify human DNA (Fig 1A and 1B). eFrog3 primers showed the greatest level of detection for bullfrog, leopard frog, and tailed frog and the weakest for clawed frog (Fig 1B). The latter introduced species demonstrates a highly restricted geographic distribution within North America and is not found in the wild in Canada. Therefore, we selected eFrog3 for further use as the frog group primer set.

### Detection Sensitivity of eDNA Assay Tools and Empirical Determination of Technical Replicate Number

Target mitochondrial gene sequences selected for eDNA assay development are expected to exist at highly diluted concentrations in large volume aquatic systems such as lakes or riverine environs. Even considering the moderate concentration factor generated by filtration and capture of total DNA material during sample preparation (~2,500 to 100,000-fold), it is crucial to develop primer sets demonstrating maximal sensitivity of detection while maintaining specificity.

It is important to note that effective characterization of these properties for each primer set demands a sufficient number of qPCR technical replicates be performed so that accurate detection frequencies can be measured, particularly at lower DNA template concentrations or when assay tools are directed towards total DNA originating from a potential confounder species where a weaker detection capability may exist.

**Fig 3:**
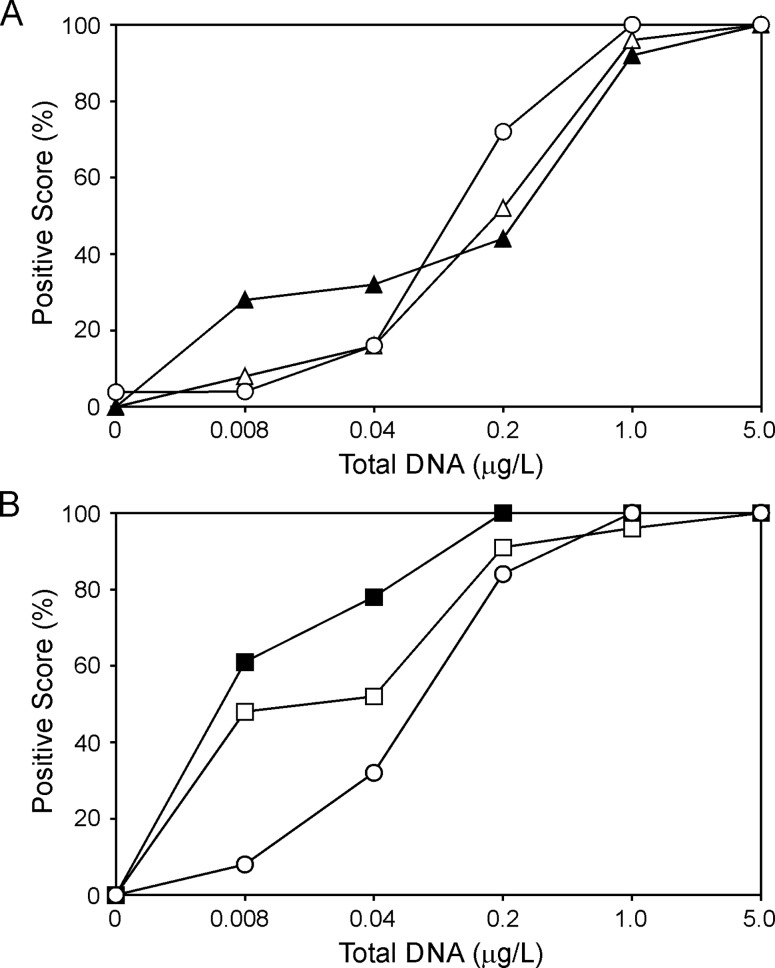
**Sensitivity of species-specific and animal group qPCR primer sets for (A) bullfrog or (B) tailed frog determined using a concentration range of total DNA.** A 5-fold dilution series (0.008, 0.04, 0.2, 1.0 and 5.0 μg/L) of bullfrog frog total DNA was assessed in the qPCR assay against eLICA1 (white triangle), eLICA2 (black triangle), and eFrog3 (white circle) primer sets in Panel A. Panel B shows the results of qPCR assays against eASMO (white square), eASMO9 (black square), and eFrog3 (white circle) primer sets using tailed frog total DNA using the same 5-fold dilution series (0.008, 0.04, 0.2, 1.0 and 5.0 μg/L). The percentage of reactions demonstrating detection following 50 cycles compared to the total reactions performed (n = 23–26 technical replicates) is shown. Negative control reactions containing no DNA template displayed no positive detection score with any of the assays with the sole exception of eFrog3 at 0.04% for the bullfrog template only.

**Fig 4:**
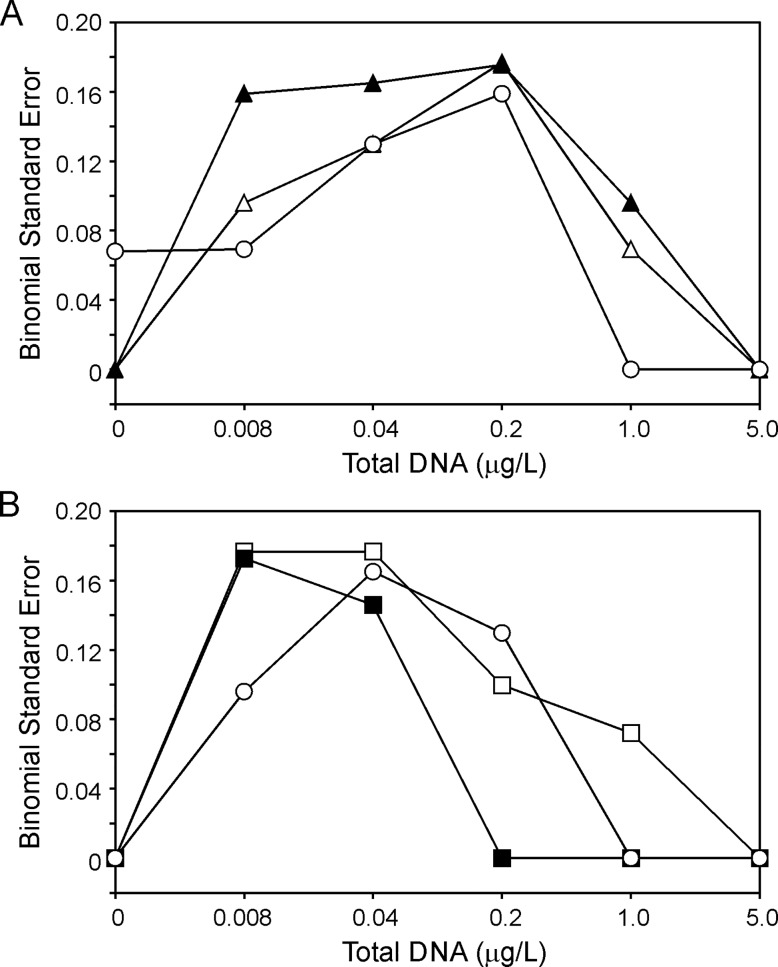
**Examples of the binomial standard error calculated across a range of bullfrog total DNA concentrations (0.008–5.0 μg/L) in a qPCR assay (n = 8 technical replicates) containing the primer sets in Fig 3 for (A) bullfrog and (B) tailed frog.**

This characteristic for eFrog3 and the bullfrog and tailed frog series of primer sets was determined using a species-matched dilution series of total DNA (0.008–5 μg/L) (Figs 3 and 4). We ran 23–26 technical replicates at each dilution for primer set assessment which then also formed the basis for determining the number of technical replicates that can be run on field samples while still having a reasonable empirically-derived standard binomial error.

In the case of eLICA1 and eLICA2, both primer sets showed a similar ability to detect the presence of their mitochondrial gene targets at higher DNA concentrations (Fig 3A). eLICA2 was more sensitive than eLICA1 with greater detection at a total DNA concentration of 8 ng/L (Fig 3A). However, this primer set was not selected for use in the field because it demonstrated reduced species-specificity compared with eLICA1 (Fig 2) which outweighs consideration of detection sensitivity. Detection of bullfrog total DNA presence was found to be equally sensitive between eLICA1 and the eFrog3 primer sets (Fig 3A).

A similar analysis was performed on eASMO and eASMO9 primer sets against a serial dilution of tailed frog total DNA. Positive detection was enhanced for eASMO9 compared to eASMO (61% *versus* 48%, respectively, at 0.008 μg/L total DNA; Fig 3B). The eFrog3 qPCR reactions on tailed frog showed similar positive detection performance to that observed for bullfrog total DNA (Fig 3A and 3B). Since species specificity was comparable between eASMO and eASMO9 (Fig 2), we selected eASMO9 due to its enhanced detection sensitivity for application to field samples.

The magnitude of binomial standard error is essentially zero at either end of the dilution spectrum (i.e. greatest confidence with high amounts of target DNA present or no DNA present; Fig 4). However, at very low concentrations, there is a higher degree of binomial standard error. To illustrate this, we can consider the binomial standard error for 8 technical replicates demonstrated in Fig 4. For each of the three primer sets tested on bullfrog, the maximal binomial standard error associated with detection was less than 0.18 across the DNA dilution series with eLICA1 (0.096) and eFrog3 (0.069) displaying reduced binomial standard error at the lowest concentrations of bullfrog total DNA examined (Fig 4A). Binomial standard error associated with the tailed frog-specific qPCR tool validation was less than 0.018 with both candidate primer sets generating similar values at 0.008 μg/L total DNA (0.172; Fig 4B). Error at this low DNA concentration was reduced for eFrog3 compared to the tailed frog-specific primer sets (0.096; Fig 4B).

Knowledge of this error is important in determining the best trade-off between choosing a reasonable number of technical replicates and the degree of confidence in the presence/”not-detected” assignation of an eDNA field sample. We therefore compared the effect that number of technical replicates has upon the binomial standard error at 0.04 μg/L total DNA (Table 3). From this analysis, we determined that running 8 technical replicates provides reasonable binomial standard error (<0.2 for all primer sets) while still being cost effective. Specifically, establishing the eDNA qPCR assay with 8 technical replicates for each sample, rather than a triplicate, allows for reduction in binomial standard error from 0.21 to 0.13 for eLICA1, from 0.24 to 0.15 for eASMO9, and from 0.21 to 0.13 (bullfrog DNA targeted) or 0.27 to 0.17 (tailed frog DNA targeted) for eFrog3 and represents a judicious compromise between operating cost and accuracy.

### Detection of PCR Amplifiable DNA Using ePlant5 Primers in Field-Collected Samples

In addition to design considerations related to species- or group-specific detection, it is important to identify and minimize any false negative data that may exist in the eDNA assay data. One important positive control is to confirm the proper assembly of qPCR components through the incorporation of separate positive control reaction through the use of an exogenous DNA template. This can be accomplished through the inclusion of a PCR amplicon, isolated total DNA containing mitochondrial DNA, or cDNA template prepared from isolated total RNA with the source material used to generate the template chosen to match the species specificity of the primer set in use [23]. To maintain reasonable PCR-clean technique during assay assembly, we support the use of the latter two positive control template sources over the use of a PCR amplicon.

Testing for the presence of substances that can inhibit DNA amplification has been suggested through spiking eDNA qPCR with exogenous DNA prior to performing assay runs [12]. However, this approach cannot assess whether the collected eDNA is insufficient to support PCR amplification due to possible issues with handling during sample processing (e.g. preservation method) or due to reduced template quality or degradation (e.g. by UV light or DNAses).

**Fig 5:**
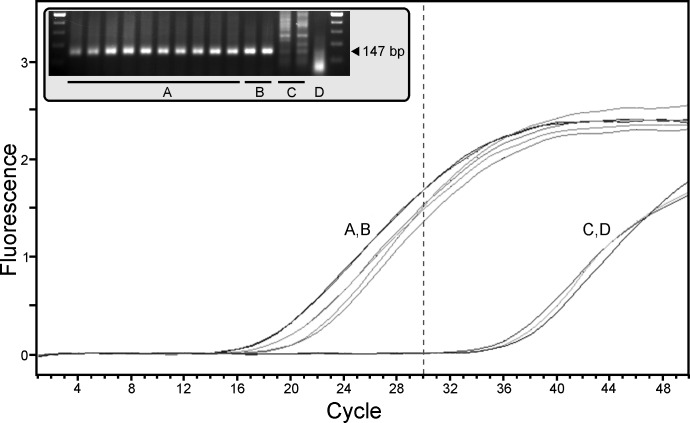
Determination of the suitability of environmental samples for eDNA qPCR assessment–Part 1 of a tripartite eDNA assay. The ePlant5 primer set was used to detect a 147 base pair region of the chloroplast 23S ribosomal RNA gene. Samples were assessed in duplicate and included 5 different environmental water sources (A), municipal tap water (B), human total DNA (C), and distilled water (D). The dashed vertical line denotes the selected data collection cut-off for use in scoring samples based upon the presence of an amplifiable environmental DNA source.

To address the requirement for a false negative control in the eDNA assay, we incorporated a component that confirms the presence of PCR amplifiable DNA material in every eDNA sample prepared from a fresh water survey location. Chloroplasts derived from plants/algae are ubiquitously found in fresh water and we designed primer sets that detect chloroplast DNA of freshwater plant species that are widespread across North America (see Table 2). Therefore use of the ePlant5 primer set confirms that recovered total DNA is of sufficient quality to evaluate further in the eDNA assay. A typical qPCR is shown in Fig 5 where the ePlant5 hydrolysis probe detects chloroplast 23S ribosomal RNA in environmental samples (A) or filtered tap water (B) early on (cycle < 30). A delayed non-specific signal can be obtained from addition of either non-target total DNA (C; 5 μg/L human DNA) or distilled water (D) into the eDNA qPCR reaction. Using this initial data and to eliminate the inclusion of false negatives in the ensuing assessment of the presence of animal-related eDNA, the ePlant5 assay was subsequently performed for 30 cycles (rather than 50 cycles) and the data scored as positive or negative for the presence of amplifiable DNA. All eDNA samples that score positive for ePlant5 would then be run in animal group and species-specific qPCR reactions for 50 cycles. In the present example of eDNA assessment of field-derived samples, we have allowed continued evaluation of any samples identified as non-assayable by ePlant5 to highlight the potential for inclusion of false negative observations in an eDNA field survey.

### Establishment of a Tripartite eDNA Assay for Field Surveys

**Fig 6:**
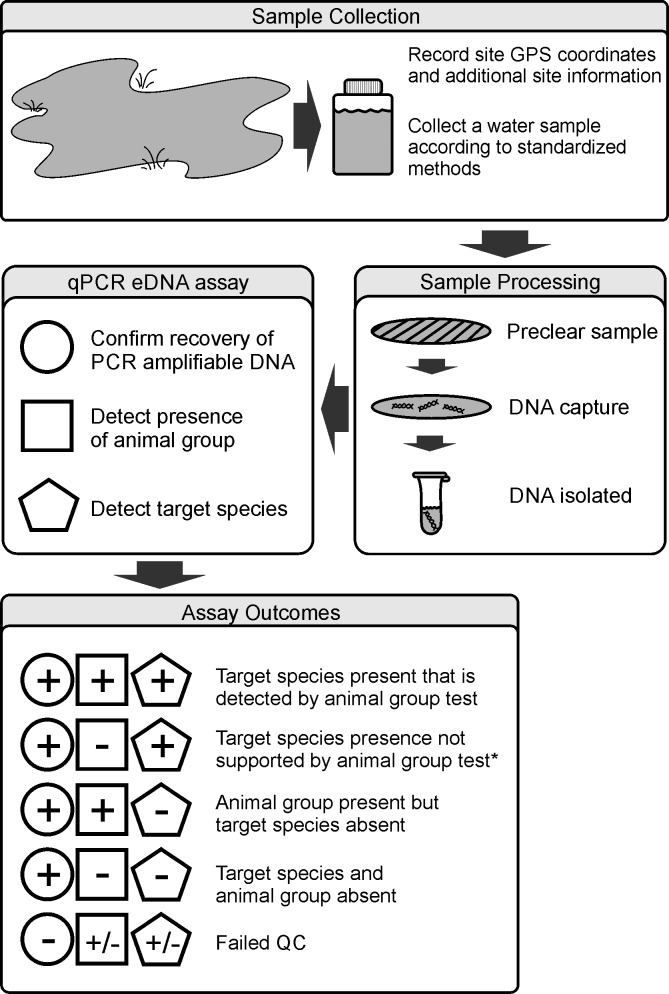
Schema depicting application of a tripartite test methodology for detection of assayable DNA (circle), animal group (square), and specific species (pentagon) within an environmental water sample to provide greater confidence in eDNA assay results. *This test result requires additional scrutiny to determine whether the negative animal group outcome is indicating a false positive species-specific detection or not.

With the different TaqMan-based primer sets validated for selectivity and sensitivity, a tripartite eDNA assay design was established that allowed for more accurate data interpretation (Fig 6). Following collection of field samples, environmental water is precleared of gross particulate matter and total DNA captured on a 0.45 μm nitrocellulose filter. This DNA material is subsequently extracted and each sample assayed for positive detection in a qPCR using the ePlant5 primer set. If the sample returns a positive score, then separate qPCR amplification reactions involving the animal group and species-specific primer sets can be performed. A negative score for ePlant5 indicates that the DNA sample is not suitable for further qPCR-based analysis. The eDNA assay, therefore, results in five possible outcomes for a given sample dependent on sample quality and primer set efficacy with respect to the particular fauna present at the sampling site (Fig 6).

**Fig 7:**
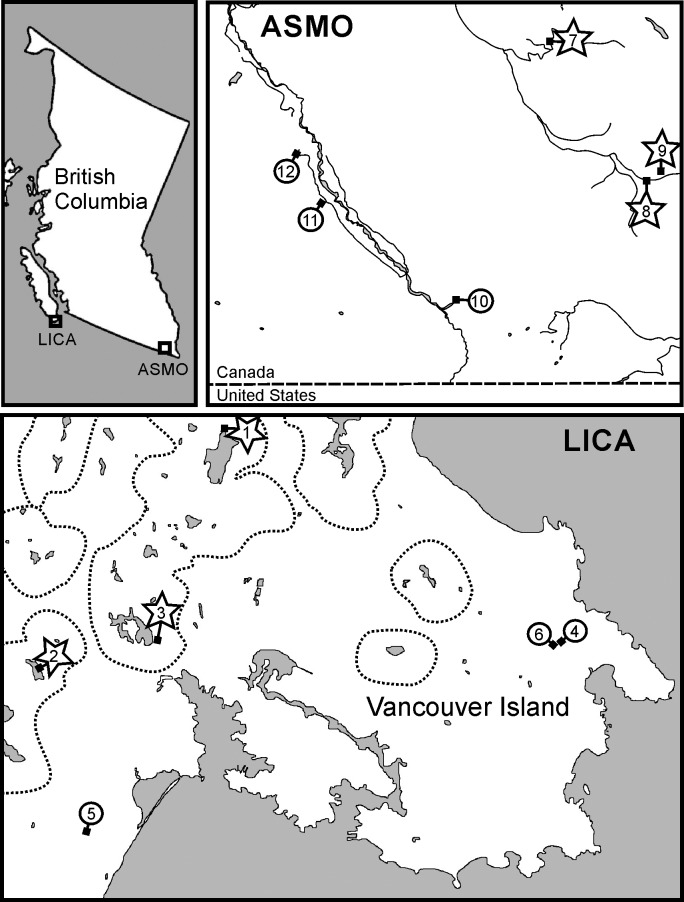
Field validation of eDNA qPCR assays directed towards bullfrog and tailed frog. Sampling sites were chosen in southern British Columbia (BC), Canada, which included known locations of habitation (star) as well as regions where the target species is absent (circle). Sites selected for validation of the tailed frog eDNA assay were located in the east Kootenay region of southeastern BC. Geographic areas across south Vancouver Island outlined by a dashed line represent locales with historical populations of bullfrog (British Columbia Ministry of Environment, BC Frogwatch Atlas (http://maps.gov.bc.ca/ess/sv/bcfa/) and BullfrogControl.com Inc (http://www.bullfrogcontrol.com/index.html).

The final phase of qPCR-based eDNA assay development involves field validation of the primer sets. With the tripartite eDNA assay assembled, we performed bullfrog- and tailed frog-associated regional surveys within south Vancouver Island and southeastern British Columbia, respectively (Table 4; Fig 7). Potential confounding variables may exist within the local environment under investigation that impinges upon DNA template performance and is not necessarily replicated by laboratory-isolated total DNA. This final field validation is essential to establish a functional scoring schema that mitigates against miscalls; the yes/no (Y/N) decision boundary for a given primer set must be determined with field samples from locations of known extant habitation (to assess false negative score rates) as well as sites where absence of the target species is confirmed (to assess false positive score rates). Six sites on Vancouver Island were visited with two 250 mL water samples collected at each locale to test for bullfrogs (Table 4; Fig 7). For tailed frog field evaluation, six sites in Southeastern British Columbia were visited with two 1000 mL water samples collected at each locale (Table 4; Fig 7).

**Table 4:** Field validation of species-specific eDNA assays directed towards bullfrog and tailed frog.

Species	Site Characteristics^a^	Site Name	UTM Coordinates (Zone, Easting, Northing)	eDNA Assay ^c^ (Amplification Frequency, Score)			Binomial Error		
				PCR Control	Group	Species	PCR	Group	Species
LICA	+, 1, 1	Prospect Lake^b^	10, 467468, 5374224	8/8, Y	8/8, Y	8/8, Y	0.000	0.000	0.000
	+, 1, 2	Prospect Lake^b^	10, 467530, 5374284	8/8, Y	8/8, Y	8/8, Y	0.000	0.000	0.000
	+, 2, 1	Florence Lake	10, 462438, 5367400	8/8, Y	3/8, Y	3/8, Y	0.000	0.171	0.171
	+, 2, 2	Florence Lake	10, 462111, 5367587	8/8, Y	3/8, Y	4/8, Y	0.000	0.171	0.177
	+, 3, 1	Thetis upper pond^b^	10, 465667, 5368089	8/8, Y	8/8, Y	8/8, Y	0.000	0.000	0.000
	+, 3, 2	Thetis upper pond^b^	10, 465668, 5368120	8/8, Y	8/8, Y	8/8, Y	0.000	0.000	0.000
	-, 4, 1	Finnerty Gardens	10, 476507, 5367602	8/8, Y	2/8, N	0/8, N	0.000	0.153	0.000
	-, 4, 2	Finnerty Gardens	10, 476507, 5367602	8/8, Y	1/8, N	1/8, N	0.000	0.117	0.117
	-, 5, 1	Murrays Pond	10, 463432, 5362205	0/8, N	0/8, N	0/8, N	0.000	0.000	0.000
	-, 5, 2	Murrays Pond	10, 463473, 5362236	8/8, Y	2/8, N	1/8, N	0.000	0.153	0.117
	-, 6, 1	Mystic Vale	10, 477246, 5367413	8/8, Y	1/8, N	2/8, N	0.000	0.117	0.153
	-, 6, 2	Mystic Vale	10, 477266, 5367383	8/8, Y	1/8, N	0/8, N	0.000	0.117	0.000
ASMO	+, 7, 1	Upper Bighorn Trib 4^b^	11, 665595, 5448057	8/8, Y	NA, NA	8/8, Y	0.000	NA	0.000
	+, 7, 2	Upper Bighorn Trib 4^b^	11, 665595, 5448057	8/8, Y	NA, NA	7/8, Y	0.000	NA	0.117
	+, 8, 1	Cabin Trib 1^b^	11, 671189, 5440590	8/8, Y	NA, NA	5/8, Y	0.000	NA	0.171
	+, 8, 2	Cabin Trib 1^b^	11, 671189, 5440590	8/8, Y	NA, NA	8/8, Y	0.000	NA	0.000
	+, 9, 1	Storm 1	11, 672736, 5441352	8/8, Y	NA, NA	8/8, Y	0.000	NA	0.000
	+, 9, 2	Storm 1	11, 672736, 5441352	8/8, Y	NA, NA	8/8, Y	0.000	NA	0.000
	-, 10, 1	Desolation 1	11, 661140, 5434077	8/8, Y	NA, NA	0/8, N	0.000	NA	0.000
	-, 10, 2	Desolation 1	11, 661140, 5434077	8/8, Y	NA, NA	0/8, N	0.000	NA	0.000
	-, 11, 1	Wigwam West Trib 1	11, 654157, 5439325	8/8, Y	NA, NA	0/8, N	0.000	NA	0.000
	-, 11, 2	Wigwam West Trib 1	11, 654157, 5439325	8/8, Y	NA, NA	0/8, N	0.000	NA	0.000
	-, 12, 1	Wigwam West Trib 2	11, 652487, 5441825	8/8, Y	NA, NA	0/8, N	0.000	NA	0.000
	-, 12, 2	Wigwam West Trib 2	11, 652487, 5441825	8/8, Y	NA, NA	0/8, N	0.000	NA	0.000

^a^Sample characteristics include location type, which denotes a site of known presence (+) or absence (-) of the target species, site number, and water sample replicate.

^b^Visual and/or auditory confirmation of the presence of adult frogs or larval tadpoles was established at this sampling site.

^c^Primer sets associated with the PCR control and animal group were ePlant5 and eFrog3, respectively. Detection of bullfrog and tailed frog species used eLICA1 and eASMO9 primer sets, respectively. Positive detection (Y) is represented by a qPCR amplification frequency greater than 2/8 while ≤ 2/8 was scored as species absent (N) from the site.

NA; not applicable. UTM, Universal Transverse Mercator.

In order to establish confidence in a positive detection, one must establish the minimum number of positive technical qPCR replicates required to support the observation. The extreme demands upon qPCR with low concentrations of DNA template and high thermocycle number (50 cycles for the frog primer sets; see Materials & Methods) in the eDNA assay renders an increased likelihood for the occasional spurious amplification and/or non-targeted release of fluor signal, even under PCR-clean conditions. To investigate this potential noise in the assay, we evaluated the results from field sites where the target species were highly unlikely to be present. We also examined filters through which only distilled water was filtered (data not shown). Based upon such replicate qPCR data for both bullfrog and tailed frog, we determined that a negative detection (N) is established when signal is detected from ≤ 2 out of 8 qPCR replicates (Table 4). Therefore, a positive species detection score (Y) would be assigned if the signal-detected qPCR replicates were greater than 2 out of 8 for a given environmental sample.

The average recovery of total DNA from a half filter following the filtration and DNA isolation procedures was 12.5 ng/μL (range 8–17 ng/μL; n = 12). The exogenous positive control present in all eDNA assay runs confirmed successful assembly and execution of the qPCR. Sites 1, 2, and 3 were independently observed to have bullfrogs present through visual and auditory observation during collections, whereas sites 4, 5, and 6 were locales where there are no known detections of bullfrog (Fig 7 and Table 4). As required for the eDNA assay, the DNA PCR amplification control (ePlant5-based test) proved positive for all samples with one exception (see Table 4). Of these, sites 1–3 tested positive for the presence of frogs (group) and bullfrogs (species) (Fig 7 and Table 4) and sites 4, 5, and 6 were negative for frogs and bullfrogs (Fig 7 and Table 4). Although the total DNA amount obtained from both replicates of site 5 were comparable (11 and 12 ng/μL), replicate 1 failed the test for the presence of qPCR amplifiable material (ePlant5 negative) and was consequently found negative for detection of animal group or bullfrog (Table 4). Although, in this instance, an appropriate lack of detection at site 5 was borne out from the replicate water sample, it is clear that without the ePlant5 evaluation, naïve incorporation of a false negative at sites 1–3 could lead to increased difficulty in scoring for the group- and species-specific evaluations. The three locations which demonstrated positive scores in the bullfrog eDNA assay are situated in regions of known extant bullfrog habitation (Fig 7).

One interesting outcome of the field validation for bullfrog relates to Florence Lake. This aquatic feature is situated in a regional control corridor established for the prevention of further spread of this introduced invasive species on southern Vancouver Island. An active removal program spanning a five year period (2007–2011) resulted in a reduction of 4,675 adult bullfrog in Florence Lake and 14,000 individuals throughout the region [24]. Our analysis in the summer of 2015 indicates that such actions failed to eliminate this species from the lake habitat (see Table 4) which is likely due to incomplete eradication and/or a recolonization of the area.

The average recovery of total DNA from the tailed frog-associated field survey was 7.3 ng/μL (range 2–14 ng/μL; n = 12). All samples prepared from sites 7 to 12 tested positive for qPCR amplification using the ePlant5 primer set and were advanced for further eDNA analysis (Table 4). The three sites of known habitation for tailed frog (confirmed visually during sampling; sites 7, 8, and 9) tested positive for the presence of the species but were negative for the frog group (Table 4, Fig 7). Sites 10 through 12, demonstrating absence of tailed frog using conventional search methods during and preceding sample collection, tested negative in the eDNA assay using both the general frog primer set (eFrog3) and tailed frog-specific primer set.

While reasonable performance of eFrog3 in detection of tailed frog DNA was noted under laboratory conditions (see Fig 1) and in the field survey for detection of the presence of bullfrog, use of this primer set for determining the presence of any frog species at the southern BC field locations demonstrated no detection data. This may be due to insufficient amplification strength by eFrog3 for certain target species when challenged with field-derived eDNA. However, it may also reflect the low likelihood of significant occupation within the southeastern BC test sites examined by other frog species as tailed frog habitat requirements exclude sympatry with other anurans; this is consistent with differences in habitat preferences of tailed frog (lotic) versus of other species (lentic/wetland). Nevertheless, further efforts are warranted to widen the utility afforded by this component of the eDNA assay

As per its original mandate, qPCR has the potential to permit quantification of the starting DNA within a sample. However, current eDNA approaches are not capable of providing a reliable quantitative measure to estimate relative abundance of target taxa at different locales despite the great interest in obtaining such information to derive indices of relative organismal abundance. This is due to potential change in known and unidentified environmental factors along with the unknown influence of several biological variables [11,13]. Environmental factors include consideration of site-specific characteristics that influence degradation of DNA in natural systems including, most notably, temperature and exposure to UV radiation. Other influential environmental variables include differing hydrological conditions between sites.

Significant biological variables impacting eDNA assay-derived determination of species abundance at survey sites include unknown distances to target taxa (i.e., point sources of eDNA), indefinite abundance of target and confounding non target taxa at a given site, and unknown emission rates (i.e. species exude DNA at different rates into the environment and, even for a given species, DNA can be released at dissimilar rates during different lifecycle phases). Moreover, a qPCR-associated estimation of animal numbers is typically derived following comparison to a standard curve generated from a known quantity of purified total DNA. However, such an estimate depends upon the amount of mitochondrial sequence disseminated into the local environment at each location under investigation. The abundance of mitochondria differs between tissue types leading to potential complexity with respect to the original source of signal (mitochondrial DNA deposition rates) between sites. For example, an anadromous salmon survey that compares sites where species-specific eDNA dissemination derives primarily from skin versus a spawning region, where dissemination could be increased from muscle tissue breakdown (during semelparity) could not necessarily be compared with the assumption that signal differences are associated solely with differing animal numbers at the two sites. The same reasoning holds true for evaluation of differing lentic sites inhabited by a given anuran species where one location contains significant egg masses compared to the other. Thus, it is important to note that the quantitative aspect of qPCR *per se* is not presently being exploited in an eDNA assay; rather the increased species specificity it affords with exploitation of the TaqMan-based format is of prime importance.

In summary, a sensibly-designed eDNA assay will have incorporated a set of steps that help characterize the nature of each sample being evaluated and allow for increased accuracy in data interpretation with minimal false assumptions playing a significant role. The work flow for a tripartite eDNA assay is depicted in Fig 6 with informed assay outcomes and we have summarized the stepwise approach in the design, validation, and execution of a qPCR-based eDNA assessment in Table 5. The current work highlights a course of assay design and validation that provides enhanced clarity in the quality and performance of qPCR-based eDNA assays thereby allowing greater context and interpretive power in their use under varied field survey conditions.

**Table 5:** Stepwise approach in the design, validation, and execution of qPCR-based eDNA assessment.

Development Phase	Step	Considerations and reporting
*In silico*	1	Available information on mitochondrial gene sequences for the target species and related potential confounder species (including *Homo sapiens*) are obtained and DNA alignments performed.
	2	Primers are designed for three eDNA assay components: (1) confirmation of the presence of assayable eDNA and subsequent detection of (2) the animal group and (3) the individual species of interest. Primer design considerations include using a 3-step thermocycle program with selection of a stringent annealing temperature for evaluation of primer sets compatible with TaqMan-based detection of DNA amplification. Sequence positions of variation between species are exploited or avoided depending upon the desired features for each assay component. Species used in design are reported.
*In vitro*	3	Each amplification primer pair is initially evaluated using species-matched purified total DNA for production of the target DNA amplicon with a reasonable level of reduced background (spurious) amplification under the extended PCR cycles required for eDNA assessment.
	4	TaqMan hydrolysis probes are subsequently evaluated for contribution to enhanced signal quality and reduced noise in a qPCR with species-matched purified total DNA.
	5	Amplification specificity is confirmed towards total DNA from the target species and against comparable related species and *Homo sapiens*. The correct identities of the DNA amplicon products from all TaqMan-based qPCR reactions are confirmed by direct sequencing or restriction endonuclease mapping.
	6	Amplification sensitivity for each primer set is evaluated with a dilution series of purified total DNA from the target species.
*In situ*	7	A tripartite eDNA assay is assembled as per the requirements of the field-based survey. This includes a no template negative control and a positive interrun control consisting of either appropriate purified amplicon template or total cDNA.
	8	Functional validation of the eDNA assay is carried out with environmental water samples collected from known positive and negative field locations for every study.
